# Long-term benefits of EPs^®^ 7630 in patients with acute sinusitis: a real-world cohort study

**DOI:** 10.3389/fphar.2024.1358879

**Published:** 2024-03-18

**Authors:** Matthias Tisch, Lubomír Roháč, Thorsten Reineke, Martin Burkart, Karel Kostev

**Affiliations:** ^1^ Department of Otorhinolaryngology, Head- and Neck Surgery, Bundeswehrkrankenhaus, Ulm, Germany; ^2^ ORL Ambulancia, Banská Bystrica, Slovakia; ^3^ Dr. Willmar Schwabe GmbH & Co. KG, Karlsruhe, Germany; ^4^ IQVIA, Epidemiology, Frankfurt am Main, Germany

**Keywords:** EPs^®^ 7630, Pelargonium, sinusitis, rhinosinusitis, nasal polyps

## Abstract

**Background:** We evaluated whether EPs^®^ 7630 prescription in patients with acute sinusitis (AS) is associated with less frequent recurrence of AS, occurrence of chronic sinusitis or nasal polyps, or fewer antibiotic prescriptions.

**Methods:** This retrospective cohort study used electronic medical records from the IQVIA Disease Analyzer database. Associations between initial therapy [EPs^®^ 7630, antibiotics, intranasal corticosteroid (INCS), or corticosteroid-free nasal spray within 3 days of AS diagnosis] and AS recurrence, incidence of chronic sinusitis or nasal polyps or rate of antibiotic prescription were studied using multivariable Cox or logistic regression models, adjusting for sex, age, insurance status, month of diagnosis, and comorbidity.

**Results:** A total of 216,360 patients were analyzed. INCS prescription was associated with a higher risk of recurrent AS (HR: 1.40; 95% CI: 1.01–1.92) and a higher incidence of chronic sinusitis or nasal polyp diagnosis (HR: 1.39; 95% CI: 1.01–1.92) compared to EPs^®^ 7630. Initial antibiotic therapy was significantly associated with higher risk of new antibiotic prescription in the period of 31–365 days after the index date compared to EPs^®^ 7630 (OR: 2.20; 95% CI: 1.66–2.92).

**Conclusion:** EPs^®^ 7630 prescription is associated with long-term benefits in AS patients. EPs^®^ 7630 can help to reduce inappropriate antibiotic use and might reduce the risk of chronic sinusitis or nasal polyps.

## Introduction

AS, also known as rhinosinusitis, is a symptomatic inflammation of the nasal cavity and the paranasal sinuses (Jaume et al., 2020; Patel and Hwang, 2021), which can be subdivided into acute (duration <12 weeks) and chronic (duration ≥12 weeks) forms under European guidelines and into acute (duration up to 4 weeks), subacute (lasting between 4 and 12 weeks), and chronic (lasting more than 12 weeks) forms under United States (US) guidelines (Rosenfeld et al., 2015; Carr, 2016; Rosenfeld, 2016; Fokkens et al., 2020).

In the literature, reported estimates of the prevalence of AS in the general population varied from 6% to 15% (U.S. Department of Health and Human Services, 2018), while a previous study from Germany reported an AS incidence of 18.8 episodes per 1,000 population per year (Hoffmans et al., 2018). Sinusitis can impair quality of life and have an impact on patient comorbidities, as well as cause increasing medical expenditure. The disease can also necessitate sick leave, which could in turn affect workplace productivity and school learning (Dykewicz and Hamilos, 2010). Most AS episodes are caused by rhinovirus, coronavirus (e.g., SARS-CoV-2) (Zou et al., 2020), influenza, parainfluenza, and respiratory syncytial virus (Rosenfeld et al., 2015; Rosenfeld, 2016; Bleier and Paz-Lansberg, 2021).

Although there are differences across the current guidelines (Aring and Chan, 2016), the available treatment options for AS include watch and wait, herbal medicine, intranasal corticosteroids (INCS), antibiotic treatment, decongestants, and nasal saline irrigations or sprays (Fokkens et al., 2020; Bleier and Paz-Lansberg, 2021). In the light of current evidence, the use of antihistamines as well as guaifenesin or glyceryl guaiacolate to treat AS is discouraged (Bleier and Paz-Lansberg, 2021).

According to the European Position Paper on Rhinosinusitis and Nasal Polyps from 2020 (EPOS 2020), current evidence also does not support antibiotic treatment for acute (post-)viral rhinosinusitis in either adults or children (level of evidence 1a) (Fokkens et al., 2020).

Medicinal products obtained from medicinal plants (phytopharmaceuticals) may be a good option for AS treatment (13). EPs^®^ 7630 is an extract from the roots of Pelargonium sidoides, drug extract ratio 1:8–10, extraction solvent: ethanol 11% (w/w). A review of the literature shows that phenolic compounds present in EPs^®^ 7630 have anti-inflammatory, antiviral, and immunomodulatory properties (Taw et al., 2013). Indeed, EPs^®^ 7630 has been demonstrated to be a safe and effective treatment for AS in adults (Bachert et al., 2009), and can therefore be considered a suitable treatment option (Timmer et al., 2013). The prescription of phytopharmaceuticals, specifically EPs^®^ 7630, has been shown to be associated with less antibiotic use and shorter sick leave duration in patients with acute respiratory tract infections (Martin et al., 2020). The European Position Paper on Rhinosinusitis and Nasal Polyps from 2020 (EPOS 2020) recommends that herbal medicines like *Pelargonium sidoides* drops have significant impact on symptoms of AS without significant adverse events (level of evidence 1b) (Fokkens et al., 2020). Nevertheless, there is still a need for real-world evidence on the use of EPs^®^ 7630 specifically in patients with AS and on its potential long-term benefits.

The aim of this study was to evaluate whether the prescription of EPs^®^ 7630 in adult patients diagnosed with AS was associated with less frequent recurrence of AS, occurrence of chronic sinusitis or nasal polyps, or fewer prescriptions of antibiotics.

## Methods

### Data Source

This retrospective cohort study was based on data from the IQVIA Disease Analyzer (DA) database, which contains case-based information provided by office-based physicians (both GPs and specialists) in Germany. The DA database includes information on patient demographics, drug prescriptions, concomitant medications, comorbidities, sick leave, and hospital referrals. The database contains data from more than 13 million patients collected between 2010 and 2020. Information is provided by nearly 3,000 office-based physicians, representing approximately 3.5% of all German practices. Practices can be categorized into ten classes based on the physician’s medical specialty (GPs and various specialists). The sample of practices included is geographically representative for Germany, covering eight major German regions.

Analyses carried out in comparison with reference statistics did not indicate any lack of representativeness or validity with respect to the DA database. The database is suitable for pharmacoepidemiological and pharmacoeconomic studies (Rathmann et al., 2018).

### Ethical aspects

German law allows the use of anonymous de-identified electronic medical records for research purposes under certain conditions. According to this legislation, it is not necessary to obtain informed consent from patients or approval from a medical ethics committee for this type of observational study that contains no directly identifiable data. Therefore, no waiver of ethical approval can be obtained from an Institutional Review Board (IRB) or ethics committee. The authors had no access to any identifying information at any time during the analysis of the data.

### Study population

This study included patients of GPs and ENT specialists diagnosed with AS (ICD-10: J01) between January 2010 and December 2020 who received a prescription of EPs^®^ 7630, antibiotics, INCS, or nasal spray without corticosteroid within 3 days of diagnosis.

**FIGURE 1:**
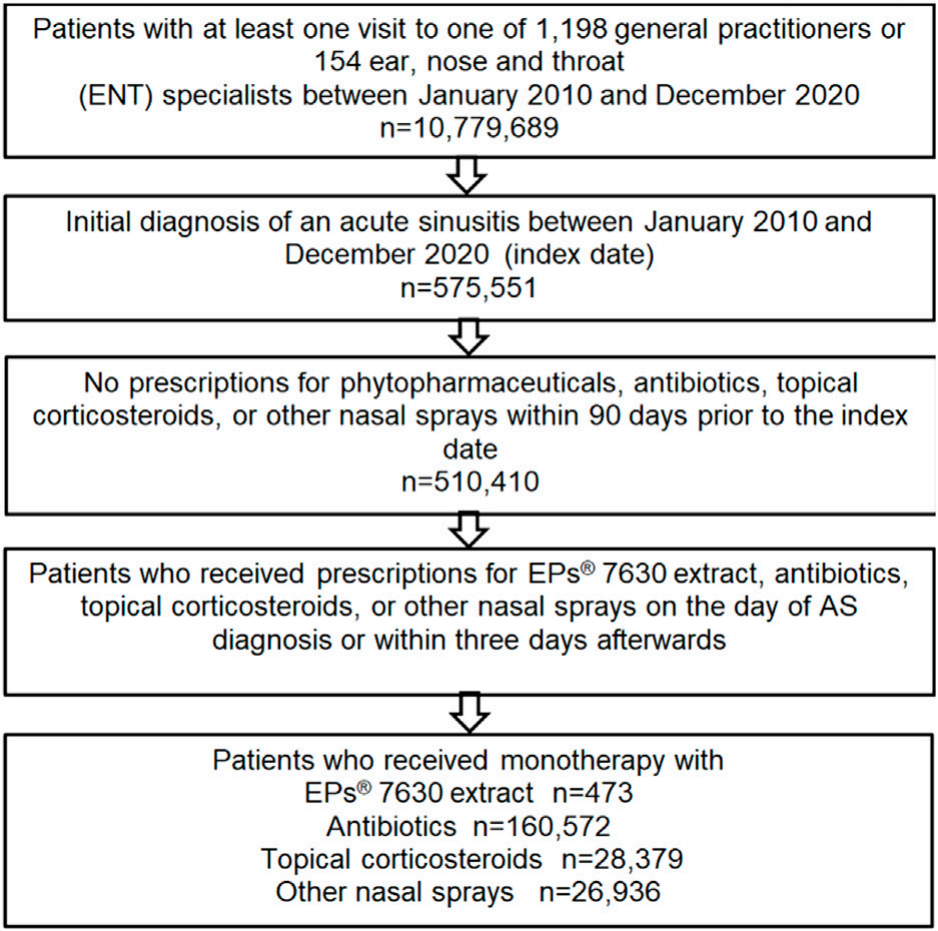
Selection of study patients.

Patients with a diagnosis of acute or chronic sinusitis or nasal polyps prior to the index date, patients with prescriptions of at least one of the above study therapies within 90 days prior to the index date, and patients receiving combinations of the above study therapies were excluded (Figure 1).

### Outcomes and covariables

The outcomes of the study were 1) percentage of AS recurrence between 31 and 365 days after the index date, 2) incidence of chronic sinusitis or nasal polyps (ICD-10: J32, J33) between 4 and 365 days after index date, and 3) percentage of patients with antibiotics prescription due to AS between 4 and 30 days and between 31 and 365 days after the index date. Descriptive statistics (mean value and standard deviation) were provided for continuous variables and the total number of patients (N) and the relative frequencies (%) for the variables. Demographic variables included age as a continuous variable, and sex, health insurance coverage (private or statutory) and Charlson Comorbidity Index (CCI) as continuous variables. CCI comprises 19 comorbidities, including diabetes with diabetic complications, congestive heart failure, peripheral vascular disease, chronic pulmonary disease, mild and severe liver disease, hemiplegia, renal disease, leukemia, lymphoma, metastatic tumor, and acquired immunodeficiency syndrome. Each comorbidity category has an associated value from 1 to 6 based on the adjusted risk of mortality or resource use, and the sum of all the values results in a single comorbidity score for a patient. A score of zero indicates that no comorbidities were found. The higher the score, the more likely the predicted outcome will result in mortality or higher resource use (Quan et al., 2005).

### Statistical methods

Multivariable Cox proportional hazard regression models were used to determine the risk of AS recurrence and the incidence of chronic sinusitis or nasal polyps while adjusting for sex, age, insurance status, month of diagnosis, and CCI. A multivariable logistic regression model was used to calculate the percentage of patients with antibiotic prescriptions due to AS also adjusting for sex, age, insurance status, month, and CCI. All of the above analyses were performed for GPs and ENT specialists separately and combined. *p*-values <0.05 were considered statistically significant. Analyses were carried out using SAS version 9.4 (SAS institute, Cary, United States).

## Results

### Baseline characteristics

A total of 132,044 patients treated by GPs and 84,316 patients treated by ENT specialists were available for analysis. Of these patients, 473 were treated with EPs^®^ 7630, 160,572 with antibiotics, 28,379 with topical corticosteroids, and 26,936 with other nasal sprays (Figure 1).

The baseline characteristics of the study patients are displayed in Table 1. The average age varied between 34.0 (SD: 18.9) years and 41.6 (SD: 17.0) years in patients treated by GPs, and between 40.5 (SD: 21.3) years and 43.7 (SD: 18.4) years in those treated by ENT specialists. Due to the young average age, most patients had a very low CCI. The proportion of men was between 40% and 44% in patients treated by GPs, and between 35% and 39% in those treated by ENT specialists.

**TABLE 1:** Baseline characteristics of the study patients.

Variable	EPs^®^ 7630	Antibiotics	INCS	Nasal spray without corticosteroid
GPs				
*N*	230	108,874	8,710	14,230
Age (mean, SD)	37.9 (16.5)	41.4 (16.9)	41.6 (17.0)	34.0 (18.9)
Male (%)	40.4	41.4	40.6	43.8
Female (%)	59.6	58.6	59.4	56.2
Privat health insurance coverage (%)	13.9	4.9	9.1	9.5
CCI (mean, SD)	0.5 (1.0)	0.7 (1.3)	0.7 (1.3)	0.5 (1.0)
December–February	35.8	32.4	36.7	36.9
March–May	25.4	27.9	24.5	27.4
June–August	13.2	14.8	11.2	7.0
September–November	25.8	24.9	27.6	28.7
ENT specialists				
*N*	243	51,698	19,669	12,706
Age (mean, SD)	41.4 (19.1)	41.2 (18.8)	43.7 (18.4)	40.5 (21.3)
Male (%)	34.6	38.3	38.6	37.9
Female (%)	63.4	61.7	61.4	62.1
Privat health insurance coverage (%)	28.8	7.1	12.1	17.8
CCI (mean, SD)	0.0 (0.3)	0.1 (0.3)	0.1 (0.4)	0.1 (0.4)
December–February	30.8	34.0	29.4	34.5
March–May	29.2	28.2	29.0	27.9
June–August	13.2	15.1	17.8	14.9
September–November	26.8	22.7	23.8	22.7

### Proportion of AS recurrence between 31 and 365 days after the index date

Table 2 shows the proportions of patients with AS recurrence between 31 and 365 days after the index date as well as the results of the regression model. While each treatment was associated with a higher incidence of recurrent AS compared to EPs^®^ 7630, the associations observed were not significant. Treatment with antibiotics or INCS was associated with an increased risk of recurrent AS compared to EPs^®^ 7630, but this association was also not significant. When patients treated by GPs and ENT practices were considered together, EPs^®^ 7630 prescription was significantly associated with a reduced incidence of recurrent AS compared to INCS.

**TABLE 2:** Association between predefined therapies and the recurrence of acute sinusitis compared to EPs^®^ 7630 between 31 and 365 days after start of therapy.

Therapy	% of patients with AS recurrence	HR (95% CI)^a^	*p*-value
GPs			
Antibiotics	10.9	1.36 (0.87–2.14)	0.176
INCS	11.5	1.43 (0.91–2.25)	0.125
Nasal spray without corticosteroid	10.6	1.28 (0.81–2.01)	0.286
EPs^®^ 7630	8.3	Reference	
ENT specialists			
Antibiotics	8.6	1.18 (0.75–1.85)	0.476
INCS	9.6	1.32 (0.84–2.08)	0.227
Nasal spray without corticosteroid	6.1	0.88 (0.56–1.39)	0.589
EPs^®^ 7630	7.8	Reference	
GPs and ENT specialists			
Antibiotics	10.2	1.25 (0.91–1.72)	0.169
INCS	10.1	**1.40 (1.01–1.92)**	**0.041**
Nasal spray without corticosteroid	8.5	1.09 (0.79–1.51)	0.585
EPs^®^ 7630	8.0	Reference	

^a^ Cox regression model adjusted for age, sex, private health insurance coverage, Charlson comorbidity index, and index month.

Bold values indicate statistically significant group differences.

### Incidence of chronic sinusitis or nasal polyps within 4–365 days after the index date

Table 3 shows the results of the chronic sinusitis or nasal polyps analysis. In both patients treated by GPs and those treated in ENT practices, we found a non-significant association between all treatment options and an increased incidence of these complications compared to EPs^®^ 7630. When patients treated by GPs and in ENT practices were considered together, EPs^®^ 7630 prescription was significantly associated with a reduced incidence of chronic sinusitis or nasal polyps compared to INCS.

**TABLE 3:** Association between predefined therapies and nasal polyps or chronic sinusitis diagnosis compared to EPs^®^ 7630 within 4–365 days after start of therapy.

Therapy	% of patients with nasal polyps or chronic sinusitis	HR (95% CI)^a^	*p*-value
GPs			
Antibiotics	6.6	1.09 (0.66–1.82)	0.729
INCS	7.6	1.33 (0.80–2.22)	0.275
Nasal spray without corticosteroid	6.3	1.10 (0.66–1.84)	0.704
EPs^®^ 7630	6.5	Reference	
ENT specialists			
Antibiotics	10.1	1.04 (0.69–1.57)	0.837
INCS	12.6	1.37 (0.91–2.06)	0.135
Nasal spray without corticosteroid	8.8	1.02 (0.67–1.54)	0.928
EPs^®^ 7630	9.5	Reference	
GPs and ENT specialists			
Antibiotics	7.7	1.07 (0.78–1.47)	0.671
INCS	11.1	**1.39 (1.01–1.92)**	**0.043**
Nasal spray without corticosteroid	7.5	1.07 (0.78–1.47)	0.682
EPs^®^ 7630	8.0	Reference	

^a^ Cox regression model adjusted for age, sex, private health insurance coverage, Charlson comorbidity index, and index month.

Bold values indicate statistically significant group differences.

### Percentage of patients with antibiotic prescription due to AS between 4 and 30 days and between 31 and 365 days after the index date

Table 4 shows results of the analysis of antibiotic prescription due to AS between 4 and 30 days and between 31 and 365 days after the index date. Depending on the index therapy, the prevalence of antibiotic therapy within 4–30 days after the start of therapy varied between 5.8% and 6.4% among patients treated by GPs and 4.4% and 7.0% among those treated by ENT specialists. Between 18.1% and 31.3% of GP patients and between 6.2% and 15.2% of ENT patients received an antibiotic prescription within 31–365 days after the start of therapy. There was no clear trend in terms of antibiotic therapy incidence in the period 4–30 days after the index date. In the period 31–365 days after the index date, however, EPs^®^ 7630 prescription was significantly associated with a lower risk of new antibiotic prescription compared to index therapy with an antibiotic in both patients treated by GPs and those treated by ENT specialists. In addition, there was a non-significant negative association between initial therapy with EPs^®^ 7630 and subsequent antibiotic prescription in the period 31–365 days after start of therapy compared to INCS (OR: 1.24 in patients treated by GPs and OR 1.50 in those treated by ENT specialists).

**TABLE 4:** Association between predefined therapies and antibiotic prescription due to AS compared to EPs^®^ 7630 within 4–30 days and 31–365 days after start of therapy (logistic regression analysis).

	4–30 days			31–365 days		
Therapy	% of patients with at least one AB prescription	Odds ratio (95% CI)^a^	*p*-value	% of patients with at least one AB prescription	Odds ratio (95% CI)^a^	*p*-value
GPs						
Antibiotics	6.4	0.77 (0.47–1.24)	0.276	**31.3**	**2.01 (1.44–2.82)**	**<0.001**
INCS	5.6	0.66 (0.41–1.08)	0.100	21.8	1.24 (0.88–1.74)	0.221
Nasal spray without corticosteroid	5.8	0.75 (0.46–1.22)	0.242	18.1	0.97 (0.69–1.36)	0.856
EPs^®^ 7630	7.9	Reference		18.3	Reference	
ENT specialists						
Antibiotics	7.0	1.42 (0.81–2.48)	0.225	**15.2**	**2.72 (1.61–4.60)**	**<0.001**
INCS	4.4	0.86 (0.49–1.50)	0.585	8.9	1.50 (0.89–2.54)	0.129
Nasal spray without corticosteroid	5.5	1.06 (0.60–1.87)	0.833	7.4	1.20 (0.71–2.04)	0.490
EPs^®^ 7630	5.4	Reference		6.2	Reference	
GPs and ENT specialists						
Antibiotics	6.6	1.01 (0.70–1.45)	0.967	**26.2**	**2.20 (1.66–2.92)**	**<0.001**
INCS	4.8	0.71 (0.49–1.02)	0.063	12.9	1.29 (0.97–1.71)	0.081
Nasal spray without corticosteroid	5.6	0.88 (0.61–1.27)	0.479	13.0	1.04 (0.78–1.38)	0.806
Eps^®^ 7630	6.6	Reference		16.7	Reference	

^a^ Logistic regression model adjusted for age, sex, private health insurance coverage, Charlson comorbidity index, and index month.

Bold values indicate statistically significant group differences.

## Discussion

In this study, based on real world data of patients treated by either GPs or ENT specialists, an initial EPs^®^ 7630 prescription was significantly associated with a reduced incidence of both recurrent AS and chronic sinusitis or nasal polyps compared to INCS when patients treated by GPs and those treated by ENT specialists were considered together. Furthermore, EPs^®^ 7630 prescription was significantly associated with a lower risk of new antibiotic prescription compared to index therapy with an antibiotic by both GP and ENT specialists in the period 31–365 days after index date.

Notably, 160,572 of 216,360 (74%) patients with a first diagnosis of AS in the present sample were treated with an antibiotic as first-line treatment. Indeed, due to the exclusion of patients receiving combination therapy, this proportion might represent an underestimation of the actual rate. In a previous unselected analysis using the same database, 52% of patients with AS treated by GPs received an antibiotic as first-line treatment (Kern and Kostev, 2021). However, current guidelines do not recommend antibiotics for this patient group: The German S2k guideline on rhinosinusitis states that antibiotics should not be given routinely for AS or acute exacerbation of recent AS and should instead only be considered for patients with specific risk factors or if there is evidence of complications (Stuck and Popert, 2017). The authors of the current Cochrane Review on antibiotics for acute rhinosinusitis in adults consider the potential benefit of antibiotics in the treatment of acute rhinosinusitis in otherwise healthy patients to be only marginal, especially when weighed against the risk of adverse effects. In addition, given the very low incidence of serious complications and due to the known issue of antimicrobial resistance, the authors concluded that treatment of uncomplicated acute rhinosinusitis with antibiotics is not justified (Lemiengre et al., 2018). Antibiotic overuse is a serious issue that is contributing to the growing problem of antibiotic resistance. Antibiotics are often prescribed for conditions that are viral in nature and will clear up on their own, as is the case with many instances of sinusitis. However, the unnecessary use of antibiotics offers no benefits and exposes patients to potential side effects (Davies and Davies, 2010; Lange et al., 2016).

Phytopharmaceuticals are non-prescription medicines in Germany. They do not have to be prescribed by a doctor and can be bought in pharmacies. However, doctors can issue a prescription to make a recommendation. Even in this case, however, patients must pay for the medication themselves as statutory insurance does not usually reimburse phytopharmaceuticals for adults. This regulation explains the small proportion of patients with phytopharmaceutical prescriptions in our study.

EPs^®^ 7630 has been shown to relieve nasal congestion and secretion and to ease pain from AS (Bachert et al., 2009). In addition, a prescription of EPs^®^ 7630 not only contributes to accelerated symptom relief, but also helps to avoid the use of antibiotics during the actual sinusitis episode: Only 6.6 percent of patients treated with EPs^®^ 7630 required an antibiotic within 30 days. This mirrors an observation made in an earlier study using data from the same database which revealed that initial treatment of an acute respiratory tract infection with phytopharmaceuticals reduced the need for an antibiotic prescription to treat the same acute infection (Martin et al., 2020). The present study adds to this finding by revealing a long-term benefit: Within 31–365 days after diagnosis of AS, initial EPs^®^ 7630 prescription was associated with a significantly lower risk of new antibiotic prescription compared to initial therapy with antibiotics. This cannot be explained by a lower incidence of chronic sinusitis or a lower AS recurrence because both rates were far below the rate of new antibiotic prescriptions and did not differ significantly between both cohorts. Both psychological and pharmacological effects may have contributed to this observation. If a GP or ENT specialist treats the first occurrence of an AS with an antibiotic, this may condition the patient to expect to receive an antibiotic for the next incidence of AS or other acute infections (Thorpe et al., 2021). Likewise, if a GP or ENT specialist observes that an AS patient recovers when treated with an antibiotic, this experience might reinforce this physician’s tendency to prescribe antibiotics (Kianmehr et al., 2020).

EPs^®^ 7630 contains a variety of pharmacologically active compounds that act synergistically to produce anti-viral, anti-inflammatory, and immunomodulatory effects (Nöldner and Schötz, 2007; Schoetz et al., 2008; Theisen and Muller, 2012). Individual polyphenols such as gallic acid revealed significant interferon-like and thus directly anti-viral and also immunomodulatory activity, enhanced the non-specific immune response, and protected host cells from lysis (Kayser et al., 2001). A recent investigation showed that EPs^®^ 7630 revealed significant *in vitro* immunomodulatory effects (Emanual et al., 2023). The immunomodulatory effects found for EPs^®^ 7630 and its compounds may help prevent excessive pro-inflammatory cytokine release and disease pathology (Nöldner and Schötz, 2007; Perić et al., 2020; Emanual et al., 2023). In patients with acute bacterial rhinosinusitis, EPs^®^ 7630 was found not only to improve symptom scores, but also to modulate the levels of individual nasal secretion of chemokines, which indicates selective immunomodulatory effects (Perić et al., 2020). The immunomodulatory properties of EPs^®^ 7630 may have contributed to a profound cure of the actual AS episode, providing a patient with effective immunity. By contrast, antibiotic treatment can impair host protective immunity to subsequent infection (Benoun et al., 2016; Kleinhenz et al., 2022).

In the present study, EPs^®^ 7630 prescriptions were significantly associated with a lower risk of recurrent AS and chronic sinusitis or nasal polyps compared to INCS. The use of INCS for the treatment of AS is a matter of debate. Current evidence has been reported to support the use of INCS as a monotherapy or as an adjuvant therapy to antibiotics for AS (Zalmanovici Trestioreanu and Yaphe, 2013). However, in a large clinical trial excluded from the Cochrane meta-analysis, a topical steroid was neither effective alone nor in combination with an antibiotic as treatment for AS in the primary care setting (Williamson et al., 2007). In the clinical practice guidelines of the American Academy of Otolaryngology—Head and Neck Surgery Foundation, INCS are only considered as an option for symptomatic relief of adult acute viral rhinosinusitis (Rosenfeld et al., 2015). The EPOS 2020 statement discourages the use of INCS for adults and children with acute viral rhinosinusitis children (level of evidence 1a) (Fokkens et al., 2020), advises prescribing INCS only when reduction of the symptoms of the acute post-viral rhinosinusitis is considered necessary, and recommends INCS for chronic rhinosinusitis. Anatomical abnormalities and smoking are the only risk factor identified to date that may predispose patients to recurrent acute rhinosinusitis. Differential effects of INCS and EPs^®^ 7630 on the local nasal immune system might have contributed to the differences observed in recurrence rates. Although allergic sinusitis should be coded as J30 according to the ICD-10 system, the code J01 used for selection of the study sample might have been used for patients with both allergic and infectious etiology. To reduce a potential bias as the result of such misclassification, we adjusted all regression analyses for month of AS diagnosis.

CRS is currently considered a syndrome with a multifactorial etiology resulting from a dysfunctional interaction between various environmental factors and the local immune system.

Epithelial barrier dysfunction is an important pathophysiological factor in CS. It is caused by defects in epithelial tight junction proteins, reduction in protective enzymes, antimicrobials, injury, etc. The transition to mature epithelial cells is disrupted, but it is not yet clear whether this is due to genetics, epigenetics, or chronic inflammatory stimulation. Barrier defects are also linked with impaired mucociliary function (Bachert et al., 2020).

In addition, barrier loss is closely linked to inflammation, as it allows the entry of antigens, irritants, and pathogens that cause inflammation, which in turn contributes to further barrier loss (Bachert et al., 2020).

A recent study found that depending on the dose administered, EPs^®^ 7630 stimulated epithelial cell proliferation, increased epithelial cell differentiation and host defense, and improved wound repair in human airway epithelial cells (Fang et al., 2023).

Depending on the cytokine profile of the inflammatory milieu (Vickery et al., 2019), CS can be distinguished into type 2 and non-type 2 CS. Type 2 CS, characterized by elevated levels of circulating and tissue eosinophils (Shah et al., 2016), appears to predominate in patients with chronic rhinosinusitis with nasal polyps (Bachert et al., 2021). However, the causes of polyp growth are not yet fully understood.

Prolonged type 2 inflammation characterized by cytokines IL-4, IL-5 and IL-13, activation and recruitment of eosinophils and mast cells are thought to contribute to the development of CRS. In Calu-3 cells infected with SARS-CoV-2, EPs^®^ 7630 reduced secretion of a range of pro-inflammatory cytokines including IL-4 and IL-13 and growth factors (PDGF, VEGF-A, CD40L) (Papies et al., 2021). A *Pelargonium sidoides*/*Coptis chinensis* extract reduced carrageenan-induced mast cell degranulation in a rat paw model (Park et al., 2018). Such immunomodulatory properties of EPs^®^ 7630 could have contributed to the reduced incidence of CRS and nasal polyps we observed.

An emerging relationship between epithelial dysfunction, type 2 inflammation, and fibrin deposition has been postulated. In a recent study, it was observed that inflammation resulted in low levels of plasminogen activator, leading to reduced fibrinolysis. This in turn caused fibrin accumulation and polyp growth (Bachert et al., 2020).

Other factors such as the presence of *S. aureus* have been suggested to potentiate certain inflammatory mechanisms of CS, contributing to polyp formation and bacterial dysbiosis (Vickery et al., 2019). Fungi are also a common trigger of chronic airway inflammation in CS and polyp formation (Tyler et al., 2021), and certain CS subtypes have shown a strong association with allergies, including allergic fungal rhinosinusitis (Marcus et al., 2018). In addition, anatomical variations are common in patients with CS (Liu et al., 2023).

Our study is a retrospective primary care database analysis with a number of limitations. First, assessments relied on ICD-10 codes entered by GPs and ENT specialists, and no diagnosis method is documented. Second, diagnosis codes do not allow for differentiation of severity levels of the diseases or the outcomes. Third, the database does not include data on the use of herbal medicines purchased by patients without prescriptions. Phytopharmaceuticals are non-prescription medicines in Germany. Patients do not need a prescription from a physician to buy herbal medicines, which are OTC drugs. However, doctors can issue a prescription to make a recommendation. Even in this case, however, patients must pay for the medication themselves as statutory insurance does not usually reimburse phytopharmaceuticals for adults. This regulation explains the small proportion of patients with phytopharmaceutical prescriptions in our study. Moreover, as patients can buy phytopharmaceuticals in the pharmacy without prescription, no data on the duration of EPs^®^ 7630 treatment is available. Fourth, no data are available on socioeconomic status and lifestyle-related risk factors (smoking, alcohol, physical activity), and the possibility of residual confounding therefore cannot be eliminated. Finally, retrospective studies do not allow conclusions to be drawn about causal relationships but instead only show associations.

## Conclusion

In this large retrospective cohort study based on data from more than 200,000 outpatients with AS, an initial EPs^®^ 7630 prescription was significantly associated with a reduced incidence of both recurrent AS and a reduced incidence of chronic sinusitis or nasal polyps compared to INCS, and with a lower risk of new antibiotic prescription compared to index therapy with an antibiotic in the period 31–365 days after index date. This study provides evidence that the initial prescription of EPs^®^ 7630 for AS is associated with significant long-term benefits for patients compared to other treatment options. Inflammation and its consequences are a key factor in recurrent polyposis. Due to its anti-inflammatory effects, EPs^®^ 7630 may play a role in polyposis patients. However, this hypothesis needs to be investigated in clinical studies as retrospective studies do not allow the conclusions on causal relationships.

## Data Availability

The datasets presented in this article are not readily available because of privacy restrictions. Requests to access the datasets should be directed to karel.kostev@iqvia.com.
